# A Review of Alternative Management Tactics Employed for the Control of Various Cockroach Species (Order: Blattodea) in the USA

**DOI:** 10.3390/insects12060550

**Published:** 2021-06-12

**Authors:** Ameya D. Gondhalekar, Arthur G. Appel, Gretchen M. Thomas, Alvaro Romero

**Affiliations:** 1 Center for Urban and Industrial Pest Management, Department of Entomology, Purdue University, West Lafayette, IN 47907, USA; thoma761@purdue.edu; 2 Department of Entomology and Plant Pathology, Auburn University, Auburn, AL 36849, USA; 3 Department of Entomology, Plant Pathology and Weed Science, New Mexico State University, Las Cruces, NM 88003, USA

**Keywords:** domestic cockroach, peridomestic cockroach, IPM, sanitation, trapping, insecticidal baits

## Abstract

**Simple Summary:**

Some cockroaches that live within and around human environments are considered pests of public health importance because they can carry and transfer human pathogenic microorganisms to food and food handling surfaces; infestations could result in cockroach allergen-induced allergic sensitization and asthma in sensitized individuals. In addition, cockroaches can cause psychological stress and stigma in people living in infested locations. Historically, cockroach control has been based on the use of insecticide sprays. However, in certain situations, sole dependence on insecticides for cockroach control can lead to issues such as pesticide resistance evolution and raises public concern due to the impact on the environment and human health. To overcome these problems, the use of reduced risk insecticide options (e.g., baits) and their combined use with alternative tactics is recommended. This review aims to examine alternative tactics used for cockroach control, with emphasis on those that are proven to be efficacious, and/or have potential for their incorporation in management programs of important domestic and peridomestic cockroaches in the USA. Remarkable examples of successful cockroach control programs are those that educate the public, promote cleanliness and hygiene, use traps to monitor infestation levels and utilize insecticide baits.

**Abstract:**

Effective control of domestic and peridomestic cockroaches requires integrated approaches that emphasize concurrent use of chemicals with alternative control tactics. An integrated pest management (IPM) approach is particularly justified in environments where satisfactory cockroach control cannot be achieved due to multiple factors including development of insecticide aversion and resistance in some cockroach species, and poor sanitation or structural issues that foster infestations. While a flurry of research effort has been devoted to study alternative tactics for cockroach control, only a few of them have been evaluated in the context of IPM programs. This review focuses on examining studies on alternative tactics that are proven efficacious, economical, and logistically feasible for their inclusion in IPM programs for important domestic and peridomestic cockroaches in the USA. Management programs that educate the public on cockroach biology, behavior, and the importance of sanitation; use of traps to monitor infestation levels; apply targeted low impact insecticides such as baits, have demonstrated a greater success for effective and sustainable control of cockroaches when compared to an insecticide-only approach. Incorporation of other alternative control methods to IPM programs will require more applied research that validates their use in real-world scenarios and demonstrates their cost-effectiveness.

## 1. Introduction

Cockroaches, which are classified in the Order Blattodea, are one of the most diverse group of insects that have inhabited our planet for at least 300 million years. Most of the approximately 4500 cockroach species that have been identified to date live in tropical and sub-tropical forests, where they primarily play a role of decomposers by feeding on dead and decaying organic matter [1]. Some species of cockroaches have adapted to living with humans since they were cave-dwellers [2]. Approximately fifty species of cockroaches are considered pests of human-made structures worldwide [3] and that number is expected to increase due to factors such as climate change and habitat destruction, including construction of human dwellings and other structures in previously forested lands. In the United States of America (USA), there are just over a dozen cockroach species that are considered pests (Table 1).

Similar to other structural insect pests, cockroaches are synanthropic pests that seek access to food, water and shelter or harborage within and around human dwellings [4]. Based on their habitat, pest cockroaches can be classified either as domestic or as peridomestic. While infestations of domestic cockroaches are mainly found in the indoor environment, peridomestic species live in the vicinity of human-made structures and enter indoors in search of nutritional resources and shelter. In addition to being nuisance pests, there are important medical implications related to cockroach infestations. For example, cockroaches can transport human pathogenic microorganisms on their bodies and physically or mechanically transfer them to food and food handling surfaces [5,6]. Similarly, infestations could result in cockroach allergen-induced allergic sensitization and asthma in sensitized individuals [7,8,9]. People who live or work in cockroach-infested structures can also experience psychological stress and social stigma [3].

Historically, cockroaches have been primarily targeted with chemical insecticides. The discovery of DDT in the 1940s, initiated an era of synthetic insecticide use for cockroach control. From 1960s to mid-1990, spray and dust formulations of insecticides from organophosphate, carbamate and pyrethroid classes were widely used for cockroach control in the USA. However, since the 1990s, sprays and dusts were mostly replaced by gel or dry bait formulations of non-repellent and slow-acting insecticides from classes such as avermectins, phenylpyrazoles, oxadiazines, and more recently insect growth regulators [10,11]. Bait formulations are easy to use, enable targeted insecticide applications, reduce human exposure to chemical insecticides and are more efficacious than sprayable insecticides [10]. Nonetheless, various over-the-counter (OTC) pyrethroid-based aerosols and “bug bombs”, which are mostly ineffective and can be harmful, are still widely used by the public as a do-it-yourself (DIY) measure for cockroach control [12].

Baits alone can drastically reduce the number of cockroaches in infested apartments, but long-term suppression of cockroach populations often requires integrating other non-chemical tactics, such as sanitation, structural modifications of buildings to prevent cockroach invasion through entry points, and continuous monitoring using insect glue boards to help inform pest control interventions [10,13,14]. Integrated approaches for cockroach control are also justified given the emergence of insecticide resistant cockroach populations, which explain control failures reported by pest management professionals (PMPs). Cockroaches, in particular the German cockroach, *Blattella germanica* (L.), have developed resistance to almost all classes of insecticides that were used for its control from the 1940s to 1990s [15].

More recently, resistance, or at least tolerance, to newer insecticides that are used in gel bait formulations has also been reported [26,27,28,29]. Adaptive physiological responses of German cockroaches are also reflected in their development of behavioral aversion resistance to sugar-containing baits (e.g., glucose, fructose, maltose, sucrose etc.) [26]. German cockroaches that are glucose-averse do not consume the toxic bait in lethal quantities and often survive insecticide exposure [30,31]. Due to these aforementioned factors, an integrated pest management (IPM) approach that emphasizes the concurrent use of chemicals with alternative control tactics continues to be considered ideal for effective control of cockroaches [13,14].

Rust [32] classified alternative German cockroach control approaches into three broad categories viz., (i) mechanical and physical, (ii) biological and (iii) novel techniques. These categories are also relevant to other indoor as well as peridomestic cockroach species. A detailed description of efficacious as well as ineffective alternative control measures can be found in review articles and book chapters by Schal [10], Gold [13], Rust [32], Schal and Hamilton [33], and Suiter [34]. Given the extensive body of literature that has critiqued alternative control tactics, the goal of this review article is to focus on alternative management strategies with reduced risk to human health and/or low or non-impact on non-target organisms that have been deployed successfully for cockroach IPM. The review also includes novel approaches that appear promising for their inclusion in cockroach IPM programs.

## 2. Domestic Cockroach Pests

This category of cockroaches includes the German cockroach and the brownbanded cockroach, *Supella longipalpa* (Fab.). Details on their taxonomic classification and basic biology can be found in Table 1. These two pest cockroach species are almost exclusively found inside homes, buildings, food handling facilities, restaurants, hospitals, animal rearing facilities, transportation vehicles, and other places. However, German cockroaches can also be found harboring outdoors during the warmer months of the year [35]. In the following sub-sections, research conducted on various alternative control measures for each of the two domestic cockroach species is described.

### 2.1. Blattella Germanica

By far, *B. germanica* is one of the most common pest cockroach species found in the USA and other parts of the world [10]. As such, a vast majority of research on alternative control measures has primarily focused on this species [13,32]. While many different forms of mechanical, physical, and biological control tactics have been studied for German cockroach control, not all of them are efficacious and/or logistically and economically practical from an IPM standpoint as described in Table 2. On the other hand, a review of recent literature on this topic suggests that practices such as (i) sanitation and public education (ii) trapping and vacuuming and (iii) use of boric acid and insect growth regulators (IGRs), are effective when combined with insecticide gel bait-based cockroach control efforts, and are detailed below. Additionally, recent advances in insecticide formulation technologies, heat treatment procedures, RNA interference (RNAi)-based gene knockdown etc., have enabled exploratory research on novel approaches for German cockroach control, which are also discussed.

#### 2.1.1. Role of Sanitation and Public Education in German Cockroach IPM

A majority of studies show positive correlation between qualitative (i.e., good, medium and poor) or quantitative (i.e., based on numerical rating) assessment of sanitation levels in human dwellings and presence of cockroach infestations [36,37,38,39]. In a survey of seven buildings, apartments with a poor sanitation rating were almost three times more likely to have an active German cockroach infestation [39]. Therefore, elimination of specific conducive conditions such as access to food and moisture sources and availability of ideal harborage places, which are collectively referred to as ‘sanitation’, are important components of a German cockroach IPM program. Indeed, several gel bait-based IPM programs that have focused on improving sanitation in infested apartments have demonstrated greater success in reducing cockroach population counts, total number of infested apartments, and allergen levels in comparison to an insecticide-only approach [37,38,39,40,41,42,43,44,45,46,47,48,49,50]. Some common examples of improved sanitation include (a) elimination of food debris or left over food in the kitchen area by means of frequent sweeping, mopping, garbage removal and dish washing, (b) minimizing cockroach access to food by storing it in airtight containers, (c) fixing leaky faucets, pipes and equipment (i.e., refrigerator, dishwasher, washing machine, etc.) to reduce water availability, (d) sealing cracks and crevices to minimize cockroach movement between apartments and limiting their access to harborage spaces in wall voids or behind cabinets, and (e) removing harborage locations such as stacks of papers, mail, grocery bags, and unused cardboard in kitchen cabinets and other locations [51].

In contrast to the findings that support the role of sanitation in the success of insecticide-based cockroach IPM programs, a few studies also demonstrate the utility of gel baits alone to reduce cockroach population numbers and/or asthma morbidity [43,52,53,54]. These results are not altogether surprising, because in certain situations the use of gel baits alone is the most effective means of controlling cockroach populations. Even PMPs seem to rely only on the use of bait formulations for cockroach control in large multi-family housing and food handling facilities. While it is logical to solely use gel baits with different active ingredients when satisfactory cockroach population reduction is consistently achieved, the incorporation of alternative measures that improve sanitation becomes necessary when infestations are not effectively suppressed by baits alone [14]. The high initial cost of implementing an IPM program for cockroach control is one of the factors that limits its adoption by PMPs. However, cost-benefit analysis suggests that long-term costs associated with IPM can be equivalent to an insecticide-based approach [37,42,45]. This is because the high initial costs of implementing an IPM program are often offset by a significant reduction in cockroach infestation rates, which minimizes the monetary input required for long-term pest control operations, especially unscheduled callbacks [37,42,45].

Deployment of successful cockroach IPM programs in housing complexes, food handling facilities, schools and other structures depends largely on cooperation from residents, employees and management staff who live or work there [38,40,43,46,47,48]. A proven method to increase resident cooperation in IPM programs is through public education [55]. Initial resident interviews that provide information on attitudes of residents towards cockroach infestations and general IPM knowledge are usually important for tailoring the educational programs to meet the needs of the target audience [39,55]. In some recent studies researchers have employed educational techniques to increase public awareness of German cockroach biology, behavior, human health risks associated with their infestations and the importance of sanitation in IPM. These educational methods can range from informal one-on-one conversations to more formal training sessions, along with distribution of relevant extension information in the form of brochures or manuals [38,40,41,43,45,46,47,48]. Using education to improve sanitation in apartments can be a slow process because residents may require enough time and help from building staff to comply with proposed recommendations [38]. However, assessing pre- and post-education sanitation levels and cockroach population numbers is a reliable way to evaluate the short-term impacts of educational programs on IPM outcomes. Statistically significant improvements in numerical pre-and post-education sanitation scores (ranging from 1 to 4 or 5) of apartments were reported in two studies that included public education as a part of their IPM program [38,43]. In the past decade, educational programs have also included information on the disadvantages and dangers of using OTC sprays, aerosols, or total release foggers by the public. More specifically, pesticide-related information included in the educational programs introduces the public to reduced risk insecticide interventions such as the use of gel baits for achieving satisfactory cockroach control [38,40,41]. Educational programs can also inform the public about the negative impacts of using OTC products on (i) efficacy of cockroach baits [56] and (ii) increase in insecticide residues in the domestic or peridomestic environments [12,38,40,48,54]. Although the direct impacts of pesticide-use education on practices against the use of OTC products has not been documented, gel bait-based cockroach IPM programs that have significantly reduced infestation rate and population numbers, also have shown a drastic decline in indoor insecticide residue levels [48,57]. It is possible that the decrease in cockroach sightings by residents or overall infestation reduction resulting from successful implementation of IPM programs discourages the use of OTC products.

#### 2.1.2. Importance of Trapping and Vacuuming in German Cockroach IPM

Typical traps used for trapping German and other species of cockroaches are made of cardboard with an interior adhesive surface that is either unbaited or baited with pheromones or food scents (e.g., peanut butter). Trapping devices used in cockroach IPM are referred to as monitors, traps, sticky traps, or glue boards depending on their trade name or user preference. The main utility of trapping cockroaches in an IPM program is for monitoring infestation levels and determining the population distribution before, during or after treatment [36,38,42,43,54,58,59]. An added advantage of using monitors in IPM programs is that it can also inform PMPs about the presence of other arthropod pests and rodents [48]. Since the distribution of cockroach infestations can vary from apartment to apartment, it is advisable to place sticky traps in areas where cockroaches are likely to have easy access to food, moisture, and harborage. Usually traps placed in the kitchen area (behind refrigerator and stove) yield a higher number of trapped cockroaches followed by pantry areas, under sink cabinets and in the bathrooms [38,47,59,60]. Traps placed in a correct location can detect previously undetected infestations of cockroaches, which is important for success of a community-wide IPM program. As an example, a survey of cockroach infestations in a multi-family housing complex revealed 118 infested apartments that were previously unreported [45]. In a decision-based [10] or assessment-based [42,54] cockroach control program, the amount and placement of baits or other insecticide interventions is based on overnight, weekly, or biweekly cockroach trap counts. Similarly, decisions on discontinuing interventions can be guided by the number of cockroaches caught on sticky traps. Generally, absence of trapped cockroaches on monitors for a continuous two- to four-week period is considered sufficient for discontinuing insecticide applications. In multi-family housing apartments where cockroach movement between apartments is common [61,62], sticky traps can be perpetually placed in apartments and inspected periodically. The advantage being that these traps can lead to early detection of population resurgence or new introductions and can thus lead to the timely deployment of interventions [45,59]. With the advent of remote monitoring technologies for rodents (ActivSense^®^, Corteva Agriscience, Indianapolis, IN, USA) and bed bugs (iMonnit^®^, South Salt Lake, UT, USA), it can be anticipated that such devices will also be available for cockroaches in the future [63]. Remote monitoring will be beneficial for early detection of cockroach infestations and reducing the labor costs associated with manual inspections of sticky traps.

A few studies have also explored the use of sticky traps alone for achieving cockroach suppression. While Ballard and Gold [64] only reported 27% cockroach population reduction after trapping ~7500 German cockroaches over two months, another study by Kaakeh and Bennett [58] observed almost 80% population reduction by trapping <1000 cockroaches in four weeks. These contrasting results likely suggest that sticky traps can lead to significant reduction in cockroach counts in places where original population numbers are low (e.g., Kaakeh and Bennett [58], but in places where starting numbers are too high, sticky traps alone may not effectively suppress cockroach populations [64]. However, in most cases, it is impossible to determine the total cockroach population numbers in any infested structure accurately. Nonetheless, the ability of sticky traps to permanently remove hundreds to thousands of cockroaches from infested apartments and their ability to inform pest management decisions based on visual counts of trapped cockroaches makes them a crucial component of an IPM program [42,43,45,59].

Another method for physical removal of cockroaches (alive or dead) from infested homes is the use of vacuums. As with sticky traps, the process of vacuuming cockroaches is viewed as an important component of the IPM process and is seldom recommended as the only tactic for cockroach control. While determining the specific contribution of vacuuming treatment in the overall cockroach population reduction within an IPM program is difficult, one study reported 80.2 and 72.5% reduction in cockroach trap catch after using flushing agent (containing pyrethins and piperonly butoxide) + vacuuming and vacuum only treatments [58]. Surprisingly the efficacy of vacuuming and vacuuming + insecticide treatments was statistically similar in the study by Kaakeh and Bennett [58], which could be likely caused by low level of overall population numbers in the housing complex. In more recent studies, anywhere between 10–3300 cockroaches were removed from 9 multi-family housing apartments (average of 300 cockroaches per apartment) by using a flushing agent + vacuuming treatment [43]. Due to its ability to remove thousands of cockroaches from an infestation, vacuuming is hypothesized to serve as an effective cockroach control strategy in large livestock barns [10]. In addition to instantaneous removal of live cockroaches and allergens from infested structures, pre-intervention vacuuming can remove food debris, dead cockroaches, egg cases, exuviae and dust in the vicinity of the bait application area, which can greatly enhance the efficacy of insecticide treatments [37,42,43]. Inclusion of vacuuming treatments in an IPM program can increase the overall costs of cockroach management [37,42,43]. However, as mentioned previously, higher initial costs of incorporating alternative methods in a control program are offset by improved efficacy resulting from IPM implementation. Due to the presence of allergens in dead cockroach body parts and dust found in infested homes that can be dispersed in the air during the process of vacuuming, the use of a vacuum with a HEPA-filter is recommended [10].

#### 2.1.3. Inorganic Insecticides and Insect Growth Regulators

Inorganic insecticides such as sodium fluoride and various forms of boron (borates and boric acid) were in wide-spread use for cockroach control before the invention of synthetic organic insecticides [10,65]. While sodium fluoride is no longer used due to mammalian toxicity concerns [10], the use of boric acid in German cockroach IPM programs has persisted for decades [10,42,45,48,49,54,65,66]. There are several factors associated with the popularity and effectiveness of boric acid for cockroach control. First, it is relatively non-toxic to humans when used as per label instructions [10] and thus it is permissible to use in locations where the use of neurotoxic pesticides is largely restricted (e.g., hospitals). Second, despite many years of use for cockroach control, there is no evidence of resistance development to boric acid [10,67]. Lastly, due to its non-specific mode of action it can be effective against cockroaches that are resistant to neurotoxic insecticides such as pyrethroids. In agreement with these perceived benefits of boric acid, several German cockroach field studies have demonstrated boric acid dust to be an important component of any gel bait-based IPM program in public housing [45,48,49]. In commercial swine farms boric acid treatments provided reduction of German cockroach population levels that were equivalent to that provided by pyrethroid insecticides [68,69]. One disadvantage of using boric acid dust is that appropriate equipment (e.g., dust applicator) is necessary for its proper application; however, without such equipment it is difficult to apply. In this regard, improper or overuse of boric acid dust can be commonly seen in infested apartments, particularly when it is used as a DIY treatment by residents. In comparison to dust formulations, gel baits containing boric acid are easy to use, but many are not as efficacious as dusts [60].

Two types of insect growth regulators (IGRs) are available for German cockroach control. These include juvenile hormone analogues (juvenoids) and chitin synthesis inhibitors (CSI or CSIs) [70]. A comprehensive description on mode of action of IGRs can be found in the book chapter by Bennett and Reid [70]. Juvenoids are not acutely lethal to German cockroaches but inhibit normal growth of immature life stages and lead to reproductive incompetence in adult insects [70]. As such, spray tank mixtures of juvenoids and neurotoxic insecticides have been used in cockroach IPM programs since the 1980s [71,72]. In addition to spray formulations of juvenoids such as methoprene, hydroprene, and pyriproxyfen, gel baits containing a mixture of neurotoxic insecticides (abamectin or clothianidin) and pyriproxyfen are now available for use in cockroach IPM. While published reports on long term efficacy of gel baits containing juvenoids for cockroach control are just beginning to become available [49,60], spray formulations of juvenoids usually show a delayed effect on population reduction that requires anywhere between 4 to 6 months or longer [70]. Another group of IGRs, i.e., CSIs, inhibit enzymes responsible for polymerization of N-acetyl glucosamine into chitin during the molting process thus leading to molting deformities and death of insects [70,73]. Several publications report significant suppression or complete control of German cockroach populations exposed to different CSIs (diflubenzuron, lufenuron, chlorfluazuron, noviflumuron etc.) in two–four months under laboratory conditions [10]. However, until recently, commercial use of CSIs for German cockroach control was almost non-existent. In laboratory tests, exposure of German cockroaches to a novel CSI, novaluron, led to suppression of population growth that was caused by death of immature cockroaches during the molting process and inhibition of fertile ootheca formation in females [74]. As of 2020, a granular bait formulation containing indoxacarb and novaluron has been introduced in the USA for the control of indoor and outdoor pests including different species of cockroaches but published data on field-level efficacy of this combination is unavailable. In general, both types of IGRs, i.e., juvenoids and CSI, fit well in German cockroach IPM programs due to their selective toxicity against insects and ability to control pyrethroid resistant cockroaches that may otherwise survive acute exposure to neurotoxic insecticides [11,70]. In this regard, there is no documented case of cockroach resistance to IGRs thus far [10].

#### 2.1.4. Emerging Technologies for German Cockroach Control

A significant amount of research effort has been devoted to identifying isolates of entomopathogenic fungi for use in German cockroach IPM. In many such studies, strains of the soil-borne fungus, *Metarhizium anisopliae* (Metchnikoff) Sorokin, have been reported to be the most virulent against German cockroaches [68,75,76]. Dual use of *M. anisopliae* with either neurotoxic insecticides or boric acid have often shown synergistic increase in cockroach control efficacy [68,76]. Although, *M. anisopliae* is not currently used for cockroach IPM, advances in formulation technology for entomopathogenic fungi have led to the introduction of a product (Aprehend^TM^, Centre Hall, PA, USA) containing *Beauveria bassisana* (Bals.-Criv.) Vuill. (another fungal entomopathogen) for the common bed bug, *Cimex lectularius* L. control [77]. This product may also provide insights for developing efficacious and safe formulations of *M. anisopliae* against cockroaches. With respect to entomopathogenic nematodes, bait stations containing juveniles of *Steinernema carpocapsae* Weiser show good efficacy against German cockroaches in the lab as well as in the field (i.e., infested apartments) [78,79]. Therefore, with improvement in the design of bait stations, it is possible to use *S. carpocapsea* for German cockroach control.

Certain plant essential oils that are derived from aromatic plants exhibit contact, fumigant and repellent activity against susceptible and resistant German cockroach populations [80,81,82,83]. Nonetheless, high volatility and short residual life of essential oils precludes their successful use in IPM. However, nano formulations of essential oils that exhibit reduced volatility and thereby prolong their residual life may promote the development and use of more effective essential oil formulations in the near future. A growing body of literature on agricultural, urban, and public health insect pests suggest that plant essential oils can be potentially used as synergists (similar to piperonyl butoxide or PBO) for enhancing the efficacy of pyrethroid insecticides particularly against resistant populations of various insect pests [84,85,86,87]. This phenomenon of pyrethroid toxicity enhancement by essential oils needs to be explored in resistant German cockroaches and offers opportunities for utilizing natural products in IPM. Lastly, various soaps and surfactants also show good activity against German cockroaches in laboratory settings [88,89]. Therefore, soaps have been proposed as a stopgap measure for cockroach control [89].

While more frequently used for bed bug control [90,91], structural heat treatment is also an option for German cockroach management. As infestations of German cockroaches and bed bugs can co-exist in certain homes [48], heat treatments targeted towards bed bugs are also likely to have some lethal or sub-lethal impacts on German cockroaches. However, formal studies to compare such adverse effects have not been conducted. Almost 25 years ago, heat treatments, an IGR (hydroprene), and vacuuming were collectively used for German cockroach control in food handling facilities at an US Army base [92]. This study is by far the most detailed record on the use of heat treatments for German cockroach control, which resulted in substantial reduction in application of insecticides in food service areas [92]. Comparison of temperature and exposure time combinations required to kill cockroaches and bed bug adults suggest that German cockroaches are more heat tolerant [90,91,92,93]. All adult German cockroaches are killed when exposed to 44 °C for 120 min, while similar mortalities can be achieved in just 25 min of exposure at 51 °C [93]. With the rise in the number of insecticide resistant German cockroach populations, Scharf and Gondhalekar [11], and Rust [32] have suggested that heat treatments should be further evaluated for inclusion in insecticide resistance management (IRM) programs.

RNA interference (RNAi) is a naturally occurring phenomenon of post-translational gene silencing in eukaryotes [94]. The discovery of RNAi has led to further investigations on utilizing it for insect pest control [95,96,97]. Crop plants expressing double stranded RNAs (dsRNAs) of insect genes have been successfully used to control corn rootworm (*Diabrotica virgifera* LeConte) [98]. RNAi is systemic in German cockroaches, which allows whole body gene silencing to be achieved by injecting dsRNA in just a few cells [99,100,101]. An approved patent from 2014, describes methods for controlling German cockroaches using dsRNA-treated food pellets [102]. Data presented in this patent shows >90% or higher mortality is achievable in three–seven weeks after initial dsRNA feeding [102]. With the availability of the German cockroach genome for identifying RNAi gene targets [100] along with rapid improvement in the dsRNA delivery technologies [98] it is likely that RNAi could be used in some form in cockroach IPM. However, it is unknown whether regulatory policies would permit the use of RNAi for indoor pest control.

### 2.2. Supella Longipalpa

Although an important indoor pest, infestations of brownbanded cockroaches are less common in comparison to that of the German cockroach. Literature on the distribution and prevalence of brownbanded cockroaches provides evidence for conflicting trends in their worldwide abundance. For example, literature prior to 2010 suggests decline in their abundance and geographic range [103,104,105] likely due to the increase in the use of indoor air conditioning systems [10]. However, reports from the past decade show that their infestations are on the rise, particularly in Asia [106,107,108,109]. Overall, brownbanded cockroaches are adapted to thrive at higher temperatures in comparison to other cockroach pest species [110]. Due to their preference for higher temperatures, brownbanded cockroaches can be found harboring in areas near the ceiling where temperatures are usually warmer as well as in heat-generating electrical equipment such as ovens, toasters, television sets, etc. [10,109,111]. Similarly, because brownbanded cockroaches, particularly nymphs, can survive for longer duration without access to moisture [112], their infestation hot spots are not limited to areas of the house or a building that are close to a water source (Table 1).

Field-based studies that collectively compare the effectiveness of different chemical and alternative methods for brownbanded cockroach control have not been conducted. However, as with German cockroaches, an IPM approach is expected to be successful in the management of brownbanded cockroach infestations [109]. Improved sanitation that reduces the availability of food, moisture and harborage can contribute to long-term suppression of brownbanded cockroaches especially when combined with insecticide baits [109]. Sticky traps and other monitoring techniques have been successfully used for assessing the abundance of *S. longipalpa* and other cockroach species [103,104,105]. In comparison to unbaited traps, supellapyrone (a sex pheromone/ attractant) baited traps were more effective in trapping brownbanded cockroaches [113,114]. The supellapyrone-baited traps are also effective in informing pest control interventions, which led to significant reduction in cockroach population numbers over time [115].

Biological control options that appear promising for the management of brownbanded cockroaches include, (i) parasitoids, (ii) entomopathogenic nematodes and (iii) entomopathogenic fungi. Two hymenopteran egg parasitoids, *Comperia merceti* (Compere) (Family: Encrytidae) and *Anastatus tenuipes* Bolivar y Pieltain (Family: Eupelmidae) have been studied for the control of *S. longipalpa* infestations [116]. Of these two egg parasitoids, *C. merceti* has been successfully utilized in student housing and research buildings for suppression of *S. longipalpa* infestations, particularly when cockroach population numbers or egg densities were high [19,117]. In laboratory studies with different cockroach species, *S. longipalpa* were the most susceptible to infection by the entomopathogenic nematode *S. carpocapsae* as indicated by lowest LT_50_ (median lethal time) values [78]. Certain strains of entomopathogenic fungi such as *M. anisopliae* and *B. bassiana* are capable of causing 80–100% mortality of brownbanded cockroaches under laboratory conditions [118,119]. In spite of the potential biocontrol options available for managing infestations of brownbanded cockroaches, PMPs will likely use only insecticide baits and liquid spray products for their control. While field studies proving insecticide bait efficacy for brownbanded cockroach control are not available, laboratory studies show that baits containing abamectin, fipronil, and hydramethylnon are efficacious against this cockroach species [120,121].

## 3. Peridomestic Cockroach Pests

In contrast to domestic or indoor cockroaches, peridomestic species are found primarily around homes, in and under landscape materials, and generally in protected areas outdoors. However, any of the species can enter homes through cracks, holes, unscreened vents, open doors and windows, particularly species that fly to lights at night. Many peridomestic species such as the American cockroach, *Periplaneta americana* (L.), smokybrown cockroach, *P. fuliginosa* (Serville), other *Periplaneta* spp., and the Turkestan cockroach, *Blatta lateralis* (Walker), can infest attics, wall voids, and crawlspaces, but there is always some access to the outdoor environment. These species can then enter indoors and be found in locations such as kitchens and bathrooms. Other peridomestic species such as the oriental cockroach *Blatta orientalis* L. may establish in crawl spaces and enter the ground floor of structures but is rarely found higher in homes. The invasive Asian cockroach, *Blattella asahinai* Mizukubo and the native *Parcoblatta* spp. may be brought into homes in bags or even on clothing, but do not establish indoors and generally are killed by desiccation.

### 3.1. Periplaneta spp. and Parcoblatta spp.

Understanding the distribution of peridomestic cockroaches outdoors leads to more localized and effective treatments. Traditional treatments for peridomestic cockroaches consisted of spraying liquid insecticides around and on the foundation of a home. This approach results in an insecticide “barrier” that is focused on preventing peridomestic cockroaches from entering homes rather than controlling the cockroaches in their habitat. The result of barrier treatments is that treated structures may not be approached by cockroaches while there is sufficient insecticide residue, but as soon as the residue has degraded (<2 weeks) [122], the home is left unprotected while the cockroach population has had additional time to increase in their harborage areas. If daytime harborages can be identified and treated, cockroach populations and reproduction can be reduced using less insecticide.

Studies by Smith et al. [123,124] provide the most detailed analysis and predictive modeling of peridomestic cockroach distribution and management. Focusing on the smokybrown cockroach Smith et al. [123] sampled insect pests at 60 homes and scored 18 house and landscape characteristics that were conducive for cockroach infestations. They further developed a cockroach habitat index model that both predicts the relative abundance of smokybrown cockroaches based on house and landscape characteristics and pinpoints high probability locations that serve as harborages and contribute to cockroach abundance. Many of the important conducive characteristics, such as house age, and type of exterior cannot be altered easily. However, other important characteristics such as number of objects (junk piles, flower planters, leaf, or pine straw litter) on a property, or tree and bush density could be altered either by removal, or by insecticide treatment. The cockroach habitat index did not estimate an exact number of cockroaches, but did estimate the relative abundance (high, medium, or low). Absolute numbers of peridomestic cockroaches vary from year to year, but some homes and properties have relatively more cockroaches than others.

Using the cockroach habitat index as a guide, Smith et al. [124] compared an IPM program based on using sanitation, landscape management, and targeted low impact insecticide applications such as baits against the conventional liquid spray barrier around homes. Implementation of the IPM program resulted in faster, longer, and better control of smokybrown cockroaches than the conventional spray treatment (and the untreated control) [124]. In a similar study, precise application of bait product was more effective than targeted sprays [122]. Further studies have shown that targeted essential oil sprays can also reduce American and smokybrown cockroach populations when integrated with cultural control measures such as sanitation and landscape management (AGA, pers. comm.). IPM programs resulted in not only superior reductions of cockroach populations, but also reduced the amount of insecticide active ingredient applied to the property by 80–95% [122,124].

Even though the focus of the cockroach habitat index and the resulting IPM program was the smokybrown cockroach, other peridomestic cockroaches co-occurred in the peridomestic environment. These species included the American, oriental, and *Parcoblatta* spp. cockroaches. Whatever treatments reduced smokybrown cockroach populations could also reduce the populations of the other species. This observation implies that the other peridomestic cockroach pest species have similar distributions within the peridomestic environment and therefore likely similar biology and physiology. Visual inspections revealed that these peridomestic pests used similar daytime harborages such as areas under rocks and planters, in utility meter boxes, and in leaf litter near trash containers.

### 3.2. Blattella Asahinai

The Asian cockroach, the phylogenetically closest species to the German cockroach, was introduced into Florida, USA in 1986 [125] and in the intervening years has spread throughout Florida and into adjacent states. It has become established in Alabama and Georgia probably by being transported on vehicles along interstates [126]. The Asian cockroach has also been reported in Texas [127], and North and South Carolina. It is estimated that this species could become established throughout the southeastern U.S. from central Texas to Georgia and as far North as Virginia.

As a peridomestic pest, the Asian cockroach is found outdoors in lawns, landscapes, and fields. It is noticeable because adults of both sexes readily fly when disturbed, and often spontaneously. Large populations can develop because they utilize leaf litter, mulch, and bushes as harborages [128,129]. Populations in southeastern Alabama increase in late May, reach their maximum in late August to early September, and then decline rapidly with cool fall weather [128]. They are usually found at the leaf litter-soil interface where the microclimate is milder and where they overwinter.

Snoddy and Appel [129] examined the mulch preferences of all stages of the Asian cockroach in the laboratory and found that adult males preferred oak leaf litter and pine straw whereas adult females preferred oak leaf litter and rubber mulch; nymphal stages preferred rubber mulch. In general, mulches that provided the most darkness and humidity were preferred. Cypress was the least preferred (and most repellent) mulch and Snoddy and Appel [129] suggested that replacing pine straw and other mulches around home with cypress mulch could reduce the number of Asian cockroaches. Cypress contains a large number of essential oil components including α-pinene, limonene, and δ-3 carene [130], all repellent and toxic essential oil components to the Asian cockroach (A.G.A., pers. comm).

A laboratory and field efficacy study of conventional insecticides and an essential oil for managing Asian cockroaches was conducted by Snoddy and Appel [131]. They examined the toxicity, repellency, and performance of β-cyfluthrin EC, fipronil granules, and an essential oil EC formulation containing 30% cinnamon and 10% thyme oil. The conventional insecticides (β-cyfluthrin and fipronil) were significantly (>25 times) more toxic than the essential oil formulation in continuous exposure and Ebeling choice box experiments. β-cyfluthrin was 100% repellent to Asian cockroaches, but both wet and dried essential oil deposits were also significantly repellent over a 7 day period. Field applications of the conventional insecticides at label rates resulted in 100% reduction of Asian cockroach populations at 7 and 30 d after treatment. The essential oil formulation was quite repellent; during application, Asian cockroaches could be seen flying and running out of the treated areas. Initially the essential oil formulation reduced populations by 68% at 7 d, but control diminished to 2% by 30 d. Therefore, the essential oil formulation had short residual activity relative to conventional insecticides. This study indicated the potential of essential oils for Asian cockroach management. Formulation and treatment optimization could potentially increase residual activity.

### 3.3. Blatta spp.

The Turkestan cockroach, *Blatta lateralis* (Walker) (Blattodea: Blattidae), is a peridomestic invasive species found in many parts of the world. The first infestations of Turkestan cockroaches in the United States were detected in 1978 at a military base in Lathrope, California and it is believed that the initial introduction occurred through infested military equipment that was brought from military bases in the Middle East and Asia [132]. Since then, the Turkestan cockroach has spread to various parts of United States [133,134,135,136,137]. The Turkestan cockroach and the Oriental cockroach have a similar biology and life cycle. One of the hypotheses about the geographical expansion of Turkestan cockroaches in the southwest is that this species has a shorter nymphal developmental period and higher production rate of oothecae, which have contributed to the displacement of oriental cockroaches in areas where they cohabit [20].

Turkestan cockroaches are well adapted to the dry climate of the southwestern United States and are often seen in underground burrows, areas with high organic matter (e.g., leaf litter, garbage), animal feed troughs, and feeding on animal manure in livestock facilities [138]. In urban settings these cockroaches establish in areas that offer hiding places (e.g., crevices around structures, manholes, crawlspaces), and moisture (e.g., sewer systems, water meter boxes, irrigation boxes) [133,136]. Although Turkestan cockroaches regularly dwell in peridomestic areas, they occasionally invade human indoor areas through holes, cracks, and gaps between door and floors [138].

Management of Turkestan cockroaches relies on the use of chemical insecticides, primarily pyrethroids that are applied in and around building exteriors [138]. Traditional perimeter application of insecticides is, however, a public concern due to the impact on the environment and potential human exposure to pesticides. Restriction on the use of insecticide perimeter treatments in California and other US states have necessitated consideration of alternative methods that supplement a PMPs toolset for Turkestan cockroach control. Environmentally sound programs for pest control are particularly demanded in sensitive environment such as public schools where traditional perimeter application of insecticides is restricted (http://ipm.ucanr.edu/PDF/PUBS/greenbulletin.2012.nov.pdf) (accessed on 10 June 2021). More integrative control approaches (i.e., IPM), that include the use of ecofriendly insecticides, sanitation, exclusion, baits, and cockroach egg parasitoids have been proposed for the effective management of Turkestan cockroaches, but the validation of these tools is just emerging [138].

Plant-derived essential oils such as red thyme oil, clove bud oil and java citronella oil have demonstrated high contact, fumigant, and repellent activity against Turkestan cockroaches [139]. Thymol, a terpenoid constituent of red thyme, exhibited the highest contact and fumigant activity on Turkestan cockroach nymphs, and was the only essential oil constituent that repelled cockroach nymphs [139]. Therefore, red thyme, clove bud, and java citronella oils, or formulations of natural product insecticides that include thymol have potential to not only kill but also repel cockroaches from points of entry into a structure or eliminate a harborage area such as a utility meter box or exert a repellent effect. Recently, fatty acids derived from coconut oil were found to exhibit high repellency activity against Turkestan cockroach nymphs (A.R., pers. comm).

Cockroach bait formulations appear to be effective against the Turkestan cockroach [137]. Laboratory work has shown that fresh or seven day-aged baits that contain active ingredients such as indoxacarb, fipronil, emamectin benzoate, or abamectin + pyriproxyfen, have high efficacy against Turkestan cockroaches. A one-year program based on outdoor application of insecticide baits supplemented with structural exclusion and habitat modification was effective at reducing Turkestan cockroaches in two areas of California [137]. In this study, significant reduction of Turkestan cockroach captures in sticky traps was observed just one month after the program was established [137]. The results of this study show that IPM programs that include baits and exclusion can help reduce Turkestan cockroach density in sensitive sites with minimal chemical inputs.

Among the possible hymenopterous natural enemies with potential use for biological control of Turkestan cockroaches are evaniid wasps, (Hymenoptera: Evaniidae) and solitary parasitoids of ootheca cockroaches (Hymenopterans: Apocrita) [140]. These wasps are frequently found attacking oothecae of cockroaches of the genera *Parcoblatta*, *Ischnoptera*, and *Cariblatta* in natural areas [141], while some studies report the presence of evaniid wasps occupying city buildings and homes and parasitizing the oothecae of oriental cockroaches [142]. Promising parasitoids that have been identified for oriental cockroach control include the eulophid wasps *Aprostocetus* (=*Tetrastichus*) *hagenowii* (Ratzeburg), (Hymenoptera: Eulophidae) due to their shorter developmental period, higher reproductive potential, and higher field parasitization rate, when compared to other naturally occurring wasps [143]. Even though evaniid wasps have been reported in urban areas of New Mexico and Arizona, identification as well as distribution, life cycles and parasitic capabilities of these wasps has not been investigated.

Several tools and strategies have been recently proposed for management of infestations of Turkestan cockroaches. Recently, laboratory studies showed that most of the liquid insecticides labeled for peridomestic cockroaches are highly effective against Turkestan cockroach nymphs when applied as residues to common harborage materials [144]. Since most of the effective insecticide formulations against Turkestan cockroaches contain pyrethroids, a known repellent chemical class [145], liquid sprays could be applied to potential harborage areas to “cockroach proof” them. Similarly, compounds with repellent properties such as essential oils, could be used around potential entry points to prevent cockroach access [146]. In contrast, application of non-repellent, toxic liquids directly to aggregations could reduce the size of the infestation without promoting cockroach dispersion to other areas. Chemical repellents can be also used in conjunction with baits in a “push–pull” control strategy [147]. This strategy has been successfully used against agriculture and livestock pests and consists of displacing insects from a resource (e.g., shelter, hidden areas) and luring them with an attractant to areas containing a pest control agent (e.g., traps, non-repellent insecticides, or baits) [148]. Successful implementation of “push–pull” control strategy for management of Turkestan cockroaches will require the identification of the most effective repellents and baits as well as a better understanding of the ecology of Turkestan cockroaches in urban settings.

Future research on IPM program for Turkestan cockroaches might also consider studies on parasitic wasps, including the identification of naturally occurring species, seasonal population dynamics, and parasitism rates. The combined use of wasps with other IPM tools could likely result in a rapid population reduction of infestations because, while targeted use of insecticides (sprays or baits) could eliminate adults and immature stages, wasps could parasitize and kill oothecae [10]. Furthermore, parasitic wasps are known to have the ability to locate various life stages including oothecae in areas where other control tools cannot be applied. However, the use of augmentative release of parasitic wasps with insecticide baits will require the selection of insecticides with minimum impact and special precautions to reduce the contact between the wasps and the baits.

## 4. Summary and Conclusions

The remarkable efficacy and convenience of bait insecticide formulations has revolutionized the approach used by PMPs for cockroach control. Most PMPs in the USA now rely on baits for cockroach control, which has drastically reduced the use of liquid spray insecticides [10]. While baits can provide satisfactory cockroach control in certain infested structures, a holistic IPM approach that combines baits and alternative interventions is ideal for long-term suppression of severe cockroach infestations [14]. Failure to adopt an IPM approach while dealing with complex pest environments can lead to perpetual cockroach infestations. Consequentially, the affected public may take the issue in their own hands and rely on hazardous OTC insecticides (e.g., aerosols and bug bombs) and other unproven alternative techniques (e.g., ultrasonic devices) for cockroach control.

Although a large number of alternative interventions have been examined for cockroach control, only a select few alternative control tactics are logistically and economically viable for inclusion in IPM programs [10,13,32,34]. Educating the public about cockroach biology and pest conducive conditions can greatly help in gaining resident cooperation for making required structural modifications (e.g., caulking crevices, fixing water leaks, repairing broken window screens etc.,) and improving sanitation in food-handling and other areas, which is key for successful deployment of bait insecticides. The use of various traps for monitoring cockroach abundance levels and hotspots within or around structures is an important IPM component that informs various pest control decisions such as choice of a bait insecticide, its quantity and placement. The use of inorganic insecticide dust (boric acid) for cockroach control has persisted for decades and even today, it is successfully used in bait-led IPM programs. With respect to IGRs, juvenoids have been an important cockroach control tool, whereas, a granular bait containing a CSI (novaluron) has been recently launched in the USA. Mass release of predators and parasitoids in the indoor environment for the control of domestic cockroach species will likely never be acceptable to the public because these biocontrol agents may themselves cause nuisance. However, the use of hymenopteran parasitoids for managing peridomestic cockroaches is a promising concept, which needs to be further explored. Technological advances in formulation technology and molecular insect pest management techniques may potentially lead to utilization of entomopathogenic fungi and nematodes and RNAi baits in cockroach IPM.

In spite of the importance of alternative interventions in cockroach control, it should however be noted that these tactics cannot be solely relied upon for successful cockroach control [13,32]. Instead, such techniques should be used as a part of an insecticide-based cockroach control approach [13,32]. While substantial funding for research on cockroach bait insecticides is mainly provided by the chemical manufacturers, there are limited competitive funding opportunities that enable in depth research on alternative control technologies [32]. Government-supported long-term investment in research that focuses on alternative control tactics for cockroach control is important because these techniques can (i) enhance the efficacy or outcomes of a bait-based IPM program, (ii) reduce insecticide use, and (iii) delay the evolution of physiological and/or bait aversion resistance in certain cockroach species.

## Figures and Tables

**Table 1:** Primary domestic and peridomestic cockroach pest species in the United States.

Type	Family	Common Name	Species	Adult Size (cm)	Development Time (Egg—Adult)	Preferred Temperature	Preferred Harborages	Flight	Notes	References
Domestic	Ectobiidae	German cockroach	*Blattella germanica* (L.)	1.3–1.6	54–215 d; mean 103 d	25–30 °C	In kitchens, near food and water, under and behind stoves and refrigerators.	No	Adult female and male macropterous; female retains ootheca until just before hatch	[16,17]
		Brownbanded cockroach	*Supella longipalpa* (Fab.)	1.0–1.5	80–124 d (33–25 °C)	25–33 °C	Throughout homes including non-food environments.	Adult males only	Adult female brachypterous; oothecal parasitoids	[18,19]
Peridomestic	Blattidae	Oriental cockroach	*Blatta orientalis* L.	2.5–3.0	180–365 d	20–29 °C	Outdoors prefer dark, moist places; utility boxes, under mulch	No	Adult female apterous, adult male brachypterous	[17]
		Turkestan cockroach	*Blatta (Shelfordella) lateralis* (Walker)	1.5–2.8	126–279 d (26.7 °C)	29–35 °C	Outdoors, in-ground containers such as water meter boxes, cable boxes, and irrigation boxes, but also hollow block walls, under broken pavement, and expansion joints	Adult males	Adult female apterous, adult male macropterous	[20]
		American cockroach	*Periplaneta americana* (L.)	2.9–5.3	150–830 d	21.1–29.4 °C	Indoors in roof voids and crawlspaces; outdoors in sewers, steam ducts, and latrines	Yes, adults of both sexes	Adult female and male macropterous	[16,17]
		Australian cockroach	*Periplaneta australasiae* (Fab.)	2.3–3.5	198–365 d (30 °C)	27–33 °C; humid	Indoors in sub-tropical areas, outdoors in tropical areas, leaf litter, under bark, greenhouses	Yes, adults of both sexes	Adult female and male macropterous	[21]
		Brown cockroach	*Periplaneta brunnea* Burmeister	3.3–3.5	263–277 d (24 °C)	24–30 °C; humid	Indoors in warm areas, outdoors in tropical areas, leaf litter, under bark, greenhouses	Yes, adults of both sexes	Adult female and male macropterous	[22]
		Smokybrown cockroach	*Periplaneta fuliginosa* (Serville)	2.5–3.8	140 (27 °C) –>634 (15 °C) d	23–30 °C; humid	Indoors in roof and wall voids and crawlspaces; outdoors in leaf litter, around trash, and in utility boxes, greenhouses	Yes, adults of both sexes	Adult female and male macropterous	[23,24]
Peridomestic	Ectobiidae	Asian cockroach	*Blattella asahinai* Mizukubo	1.3–1.6	45 (30 °C)–123 (20 °C) d	22–30 °C	Compost, leaf litter, grass, and pine straw	Yes, adults of both sexes; strong flyer	Active throughout the day and night. Huge populations may develop	[25]
		Wood cockroaches	*Parcoblatta* spp.	1.2–3.1	284–383 d 1–2 generations per year	15–28 °C	Leaf litter, compost, under rocks and fallen trees	Adult males	Adult males macropterous, females apterous or brachypterous	[16]

**Table 2:** A list of alternative German cockroach control strategies that are not currently used in IPM programs. See Schal [10], Gold [13], and Rust [32] for a detailed description of these alternative control measures.

Alternative Control Measures	Reasons for Non-Use
Predators	Nuisance to homeowners, fear, may bite
Parasitoids	Possible nuisance, long life cycle, host specificity issues
Modified atmosphere treatments (ozone and low oxygen treatments)	Not economically practical, require extensive preparation and long exposure times
Sterilizing agents (other than insect growth regulators)	Potentially toxic to humans and pets
Electromagnetic and ultrasonic devices	Are neither perceived by or toxic to cockroach pest species
Low temperatures	Extended periods would not be acceptable to residents, cost prohibitive
